# Genetic control of the lateral petal shape and identity of asymmetric flowers in mungbean (*Vigna radiata* L.)

**DOI:** 10.3389/fpls.2022.996239

**Published:** 2022-09-29

**Authors:** Xin Li, Mingzhu Sun, Yahui Jia, Dan Qiu, Qincheng Peng, Lili Zhuang

**Affiliations:** ^1^ College of Life Science, Nanjing Agricultural University, Nanjing, China; ^2^ School of Life Sciences, Sun Yat-Sen University, Guangzhou, China; ^3^ College of Agro-grassland Science, Nanjing Agricultural University, Nanjing, China

**Keywords:** Fabaceae, *Vigna radiata* L, flower asymmetry, lateral petal identity, VrCYC3

## Abstract

Broad diversity of flowers in Fabaceae provides a good system to investigate development and evolution of floral symmetry in higher plants. Many studies have demonstrated a conserved mechanism controlling development of zygomorphic flower during last decades. However, the molecular basis of how asymmetric flower established is largely unknown. In this study, we characterized mutants named *keeled wings* (*kw*) in mungbean (*Vigna radiata* L.), which is a legume species with asymmetric flowers. Compared to those in the wild type plants, the lateral petals were ventralized in the *kw* mutants. Map-based cloning showed that *KW* was *VrCYC3* gene in mungbean, the ortholog of *Lotus japonicus CYC3* (*LjCYC3*) and *Pisum sativum CYC3* (*PsCYC3*). In addition, another two *CYC*-like genes named *VrCYC1* and *VrCYC2* were identified from mungbean genome. The three *CYC*-like genes displayed distinct expression patterns in dorsal, lateral and ventral petals. It was found that VrCYC3 was located in nucleus. Further analysis showed that VrCYC3 had transcription activity and could interact with VrCYC1 and VrCYC2 in yeast cell. Moreover, the deletion of two amino acid residues in the R domain of VrCYC3 protein could decrease its interaction with VrCYC1 and VrCYC2 proteins. Our results suggest that LjCYC3/VrCYC3 orthologs play conserved roles determining the lateral petal shape and identity of zygomorphic flower as well as asymmetric flower in Papilionoideae.

## Introduction

Floral symmetry is a distinct character with diversity in flowering plants, with three main types including actinomorphy, zygomorphy and asymmetry (Endress, 1999; Citerne et al., 2010; Hileman, 2014). Multiple studies have revealed that evolution of floral symmetry cooperates with pollinators to improve the plant productivity (Endress, 2001; Cubas, 2004; van der Kooi et al., 2021). It has been shown that *CYCLOIDEA* (*CYC*) and its paralog *DICHOTOMA* (*DICH*), encoding TCP transcription factors, are involved in the control of zygomorphic flower development in *Antirrhinum majus* (Luo et al., 1996; Luo et al., 1999). Furthermore, it has been found that CYC-like TCP family proteins have been recruited to play central roles in determining zygomorphy development in different species (Howarth and Donoghue, 2006; Busch and Zachgo, 2007; Broholm et al., 2008; Kim et al., 2008; Hileman and Cubas, 2009; Jabbour et al., 2009; Preston and Hileman, 2009; Rosin and Kramer, 2009; Zhang et al., 2010; Fambrini et al., 2011; Busch et al., 2012; Yang et al., 2012; Yang X. et al.,2015; Citerne et al., 2017; Dong et al., 2018; Hsu et al., 2018; Spencer and Kim, 2018; Zhao et al., 2018; Sengupta and Hileman, 2022; Sun et al., 2022).

Fabaceae is the third largest family of plants, with more than 600 genus and 18000 species (Graham and Vance, 2003). The broad diversity of flowers in Fabaceae provides a good system to investigate the development and evolution of floral symmetry. Three *CYC*-like TCP genes, belonging to the CYC2 clade of ECE TCP genes (Howarth and Donoghue, 2006), with overlapping and divergent functions in the establishing dorsal-ventral patterning (three types of petals: dorsal petal, lateral petal and ventral petal) have been identified in Papilionoideae species with zygomorphic flowers including *Lotus japonicus* and pea (*Pisum sativum*) (Citerne et al., 2003; Citerne et al., 2006; Feng et al., 2006; Wang et al., 2008; Woollacott and Cronk, 2018; Zhao et al., 2019). For example, *LjCYC2/PsCYC2* interacts with *LjCYC3/PsCYC3* to confer dorsal petal identity in *L. japonicus* and pea, while *LjCYC3/PsCYC3* controls the lateral petal identity (Feng et al., 2006; Wang et al., 2008). The lateral petals in flowers of *ljcyc3* and *pscyc3* mutants are ventralized, displaying the shape of the ventral petals in the wild type plants (Feng et al., 2006; Wang et al., 2008).

**Figure 1:**
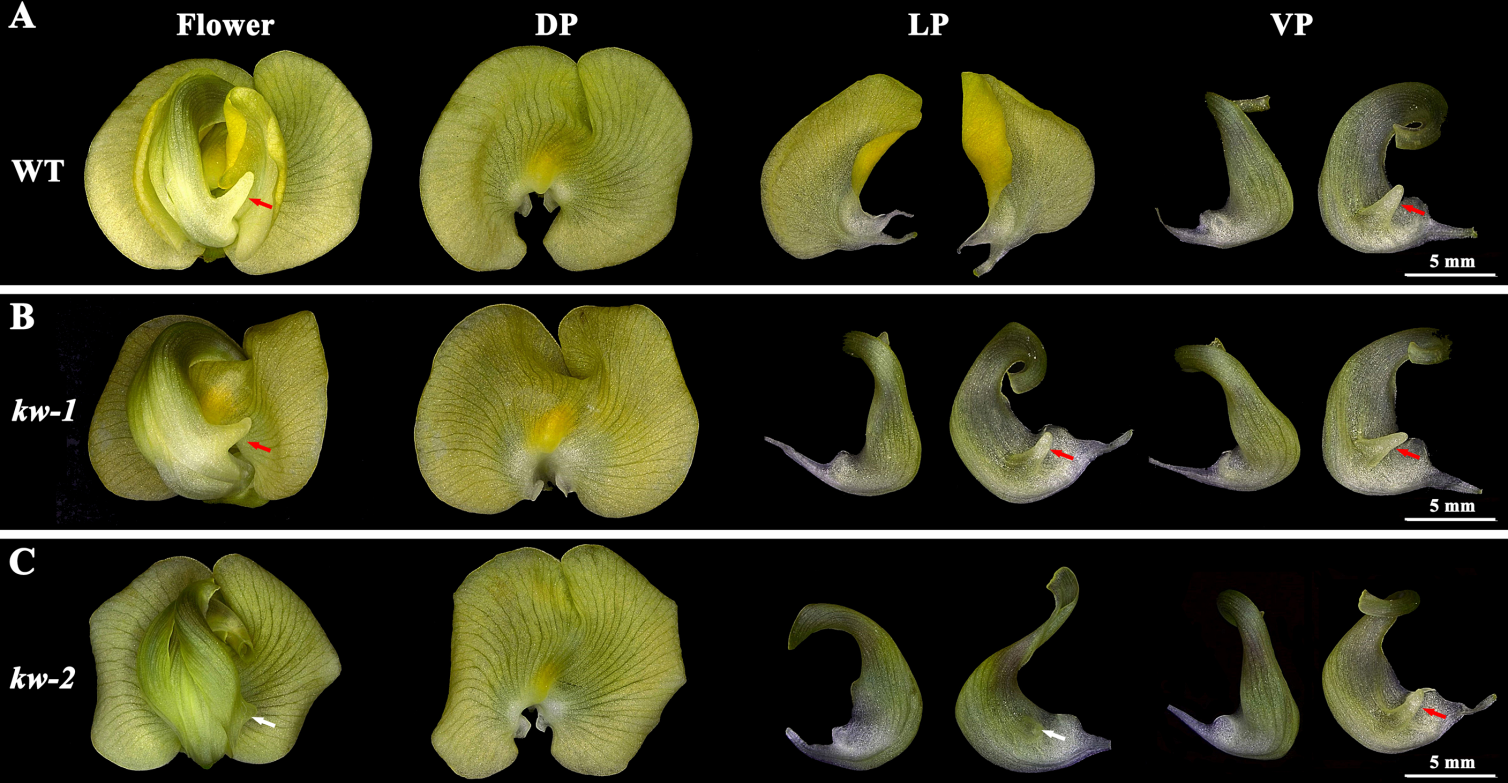
The floral phenotype of WT and *kw* mutants. Petals of the flowers in the wild type of plant **(A)**, *kw-1* **(B)** and *kw-2* **(C)** mutants possessed dorsal-ventral (DV) differentiation. DP, dorsal petal; LP, lateral petal; VP, ventral petal. Red arrows indicated petal spurs. White arrows indicated retarded petal spurs.

However, unlike most Papilionoideae species with zygomorphic flowers, some lineages including many species in *Vigna* genus such as mungbean (*Vigna radiata*) and *V. caracalla* have asymmetric flowers (Endress, 1999; Etcheverry et al., 2008; Guo et al., 2019). For example, in mungbean flower, the dorsal petal unfolds, right lateral petal is closely attached to ventral petals, the tip of left lateral petal curls inward, two ventral petals fuse together, and a conspicuous spur develops on the left ventral petal (**Figure 1A**). Interestingly, the three types of petals are internal asymmetric and maintain the distinct dorsal-ventral patterning in these species. But, the underlying mechanism is largely unknown in the asymmetric flower.

In this study, we identified two allelic mutants, *keeled wings* (*kw*) in mungbean, exhibiting the deficiency in lateral petal identity. Map-based cloning showed that KW was LjCYC3 ortholog in mungbean. Our results suggest that LjCYC3/VrCYC3 orthologs play conserved roles in determining lateral petal identity of flowers with zygomorphy as well as asymmetry.

## Materials and methods

### Plant materials

The mungbean *kw* mutants (*kw-1* and *kw-2*) were identified from the gamma ray mutagenesis population under the accession Sulu (Jiao et al., 2019). For phenotype analysis, WT plants and *kw* mutants in greenhouse were grown at 30/28°C (day/night temperature) with 16 h photoperiod of 250 μmol m^-2^ s^-1^ photosynthetically active radiation. The allelic tests for two mutants were carried out by crossing the *kw-1* mutant with the *kw-2* mutan. Ten plants of F_1_ generation showed the mutated phenotype.

### Molecular cloning of the *KW* gene

Genetic mapping of *KW* in mungbean was conducted as described previously (Jiao et al., 2016). We crossed the *kw-1* mutants with another accession AL127 and the phenotype of F_1_ plants were normal. The *kw-1* mapping population (F_2_ population) was constructed from the self-pollination of F_1_ flowers. The 265 individual plants with mutant flower phenotype were identified in the F_2_ population with a total of 1150 plants. For fine mapping of *KW* gene in mungbean, new markers were developed according to InDels information identified from genome re-sequencing between the accessions AL127 and Sulu (Jiao et al., 2016). Primers used for genetic mapping and gene cloning were listed in **Supplementary Table 1**.

### Scanning electron microscopy

The samples of inflorescence and floral buds were fixed with FAA and then dissected to reveal the internal floral organs. The samples were dehydrated in an alcohol series. The samples for SEM analysis were prepared as described by Chen et al. (2002). The scanning electron microscopy (SU8010, Hitachi, Tokyo, Japan) analysis was conducted as previously described (Jiao et al., 2019).

### Quantitative reverse transcription PCR

Total RNA was isolated from dorsal, lateral and ventral petals from the flowers at development stage 9 of mungbean following manuals (Omega, Shanghai, China). First strand cDNA was synthesized *via* Takara PrimeScript™ RT reagent Kit (TaKaRa, Dalian, China) after removing genomic DNA. qRT-PCR analysis was conducted using TB GreenTM Premix ExTM RR420A (TaKaRa), and on the ABI StepOnePlus machine (Applied Biosystems, Foster City, CA, USA). The amplification condition was set as following: initial denaturation at 95°C for 10 min, followed by 40 cycles of 95°C for 15 s, 60°C for 15 s, and 72°C for 20 s. Three biological replicates with three technical repeats were conducted. *VrTUB* was selected as the reference gene in mungbean (Jiao et al., 2019). Relative gene expression level was determined using the 2^−ΔΔCt^ method (Livak and Schmittgen, 2001).

### Subcellular localization of VrCYC3 protein

For analysis of the subcellular localization of VrCYC3, the coding sequence (CDS) of *VrCYC3* was amplified by PCR method and inserted into the pA7-YFP vector. Mungbean protoplasts were isolated and DNA-polyethylene glycol (PEG)-calcium transformation was conducted following the protocol as described by Li et al. (2019). The transformed protoplasts were observed and photographed by the confocal laser scanning microscopy (Leica, TCS SP5, Wetzlar, Germany).

### Yeast two-hybrid assay

The yeast two-hybrid (Y2H) assays were carried out following the protocol (Clontech). The coding sequences of *VrCYC1*, *VrCYC2*, *VrCYC3* and the mutated form *VrCYC3m* with 6-bp deletion (at the position 174-178 of *VrCYC3* CDS) were amplified and cloned into the pGADT7 and pGBKT7 vectors respectively. The constructs of AD and BD fused to the VrCYCs were co-transformed into AH109 cells. The clones were screened on the plates with selective media SD/-Leu-Trp and SD/-Leu-Trp-Ala-His with 80 mg/mL X-α-gal.

### Phylogenetic analysis of TCP family proteins

The TCP family proteins in mungbean, *L. japoniucs* and *Arabidopsis thaliana* (**Supplementary Table 2**) were aligned *via* MEGA 7.0 (Kumar et al., 2016). The phylogenetic tree was built by the neighbor-joining method with 1,000 bootstrap replicates and performed by iTOL v6 (https://itol.embl.de).

## Results

### Characterization of *keeled wings* mutants in mungbean

In order to investigate the genetic mechanism of asymmetrical flower development in mungbean, screening for variations of flowers and petals was carried out on a large-scale gamma ray mutagenesis population about 70,000 M_2_ lines (Jiao et al., 2019). We identified the *keeled wings* (*kw*) locus in mungbean with two mutated alleles (*kw-1* and *kw-2*) (**Figure 1**), affecting dorsal-ventral patterning during flower development. In *kw-1* mutant, there were ventralized petals at the lateral position and the left lateral petal possessed a spur (**Figure 1B**), which developed normally on the left ventral petals in the wild type plants (**Figure 1A**). While, in *kw-2* mutant, the ventralization was somehow partial and a retarded spur developed on the left lateral petals (**Figure 1C**). No other obvious phenotype was observed in dorsal and ventral petals in the *kw* mutants, compared with the wild type plants.

**Figure 2:**
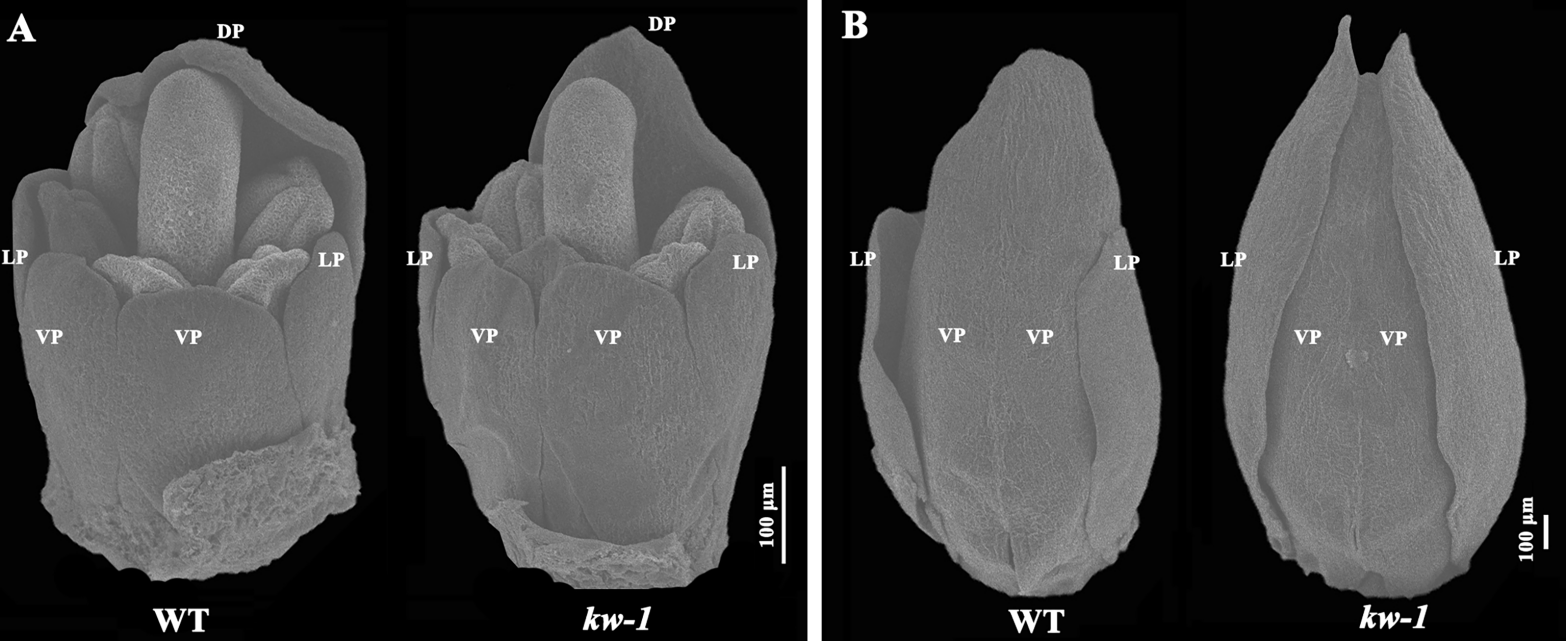
SEM analysis of flower development in WT and *kw* mutant. Floral organ development at the flower development stage 8 **(A)** and the stage 9 **(B)**. DP, dorsal petal; LP, lateral petal; VP, ventral petal.

Using scanning electron microscopy, flower development process was observed and compared between the wild type plants and mutants (**Figure 2**). It was found that the length of the lateral petals was similar to that of the ventral petals at the developmental stage 8 (Tucker, 2003), and there was no obvious difference between the wild type of plant and *kw* mutant at this stage (**Figure 2A**). However, at stage 9, when the fused ventral petals enclosed the stamens and carpels, the length of the lateral petals in the wild type of plant was two-thirds of that of the ventral petals, but the lateral petals in *kw* mutant mimicked that of the ventral petals (**Figure 2B**).

### Genetic mapping of *KW* in mungbean

Genetic analysis of *kw* was carried out by backcrossing the *kw-1* mutant with its wild type of plant, Sulu. The flowers of F_1_ plants displayed a normal phenotype. An F_2_ population consisting of 114 plants was constructed to investigate the segregation ratio. 88 individuals displayed the wild type flowers, and 26 individuals exhibited the mutant flowers. Thus, a segregation ratio of 3:1 (χ^2^= 0.29<χ^2^_0.05_ = 3.84) confirmed that *kw* mutant was controlled by a single recessive gene in mungbean.

**Figure 3:**
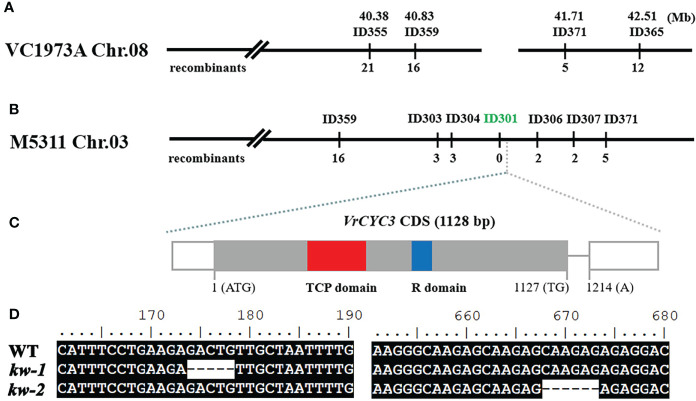
Genetic mapping the *KW* gene. **(A)** Genetic map of the *KW* gene on chromosome 8 in mungbean VC1973A genome; **(B)** Fine mapping of *KW* based on the mungbean M5311 genome. The molecular marker ID301 was co-segregated with the *kw-1* mutant phenotype in the F_2_ mapping population. **(C)** Schematic diagram of the *VrCYC3* gene. Exons, the rectangles; Intron, the line. The CDS in exons were highlighted in grey and the non-coding sequences in white. **(D)** Mutations in ORF of *VrCYC3*. Alignment of *VrCYC3* coding sequences from WT, *kw-1* and *kw-2*. Numbers on the top of the sequences indicated the position on the open reading frame.

In previous study, we have developed a set of insertion/deletion (InDel) markers to map genetic loci controlling the traits in mungbean (Jiao et al., 2016). Using the F_2_ mapping population from the cross between *kw-1* and AL127, *KW* was located on chromosome 8 (Chr.08) of VC1973A genome (Kang et al., 2014). By chromosome walking approach based on developing molecular markers (**Supplementary Table 1**), *KW* was narrowed down to a region located at 40.8 Mb and 42.17 Mb between the markers ID359 and ID371 (**Figure 3A**). However, it was found that there was a big gap in the *KW* mapping region of mungbean VC1973A genome, based on the comparative analysis with aduzki bean (*Vigna angularis*) genome (**Figure 3A**; Yang K. et al., 2015). Recently, by combining short-read, long-read, and high-throughput chromatin conformation capture (Hi-C) sequencing technologies, we generated a chromosome-level genome of mungbean M5311 (data not shown). Thus, we analyzed the corresponding region on the chromosome 3 of mungbean M5311 genome to clone the *KW* gene (**Figure 3B**). Finally, *KW* was narrowed down to a 240 kb interval between the markers ID303 and ID306 (**Supplementary Table 1**), and the marker ID301 was co-segregated with the *KW* gene in 265 mutated plants out of a total of 1150 individuals from the F_2_ mapping population (**Figure 3B**).

### *KW* was the ortholog of *L. japonicus LjCYC3* in mungbean

**Figure 4:**
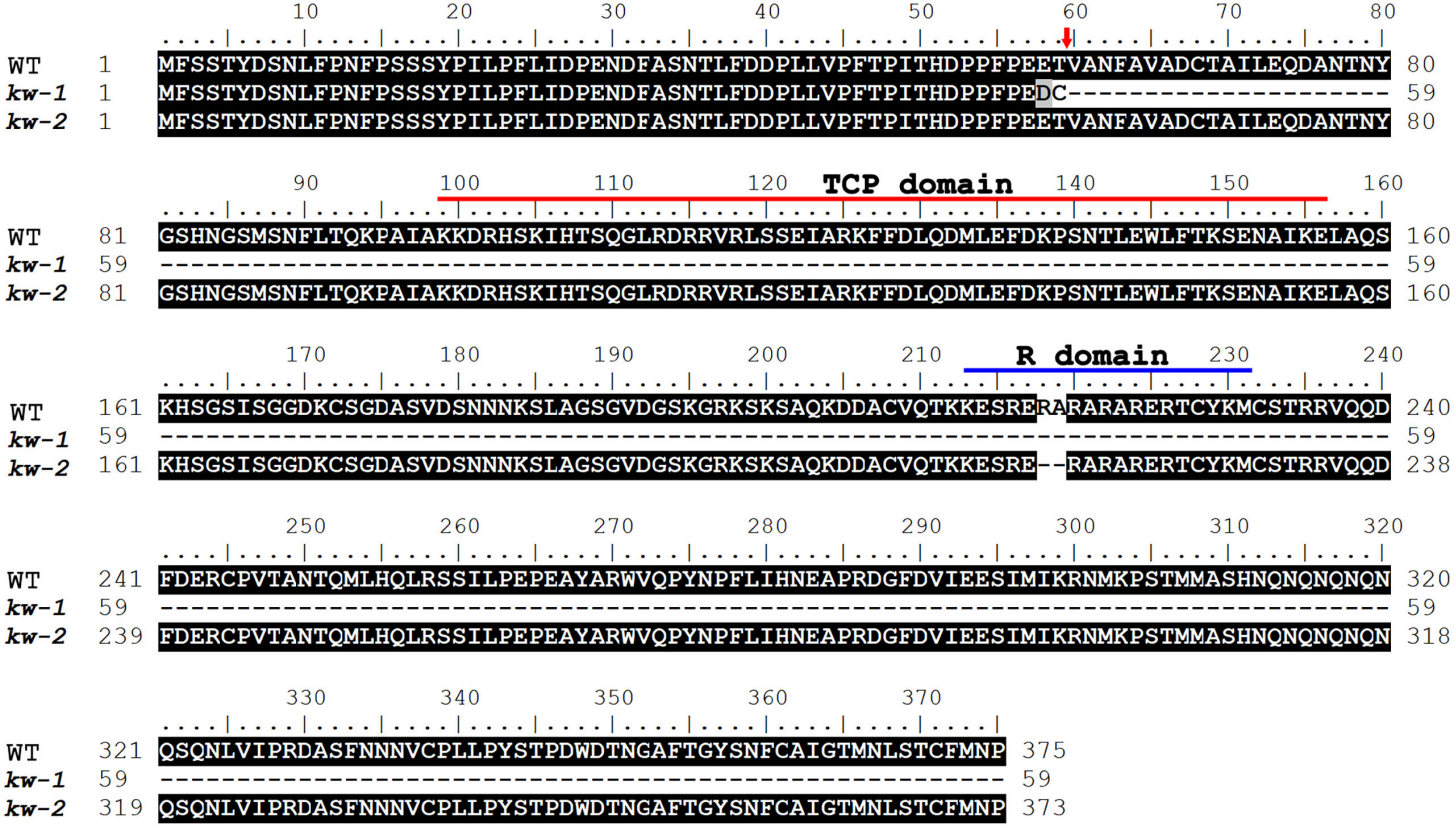
The predicted sequences of VrCYC3 and its mutations in mungbean. Alignment of the VrCYC3 protein sequences from the wild type plant (WT), *kw-1* and *kw-2* mutants. Red line, TCP domain; Blue line, R domain; Red arrow indicated the truncated site in the *kw-1* mutant.

In the *KW* mapping interval, there were 25 putative genes (**Supplementary Table 3**). One of the candidates (*VradChr3T0011489.1*) encoded a TCP family protein which was the ortholog of *LjCYC3* and *PsCYC3* in mungbean (**Figure 3C**). Combined with the similar mutant phenotype, we assumed that *VradChr3T0011489.1* (*VrCYC3*) was the likely candidate for KW in mungbean. When the sequences of *VrCYC3* were analyzed, different deletions were found in both *kw*-*1* and *kw*-*2* mutants (**Figure 3D**). A 5-bp deletion was detected at the position 174-178 of the *VrCYC3* ORF (Open Reading Frame) in *kw-1* mutant (**Figure 3D**). It maycause a frameshift and premature stop codon, thus resulting a truncated protein that lacking most parts of VrCYC3 protein (**Figure 4**). In addition, a 6-bp deletion at the position 668-673 of the *VrCYC3* ORF was detected in *kw-2* mutant (**Figure 3D**), which would cause the translation of a mutated protein, lacking two amino acid residues, Argnine (R) and Alanine (A), in the R domain (**Figure 4**; **Supplementary Figure 1**).

**Figure 5:**
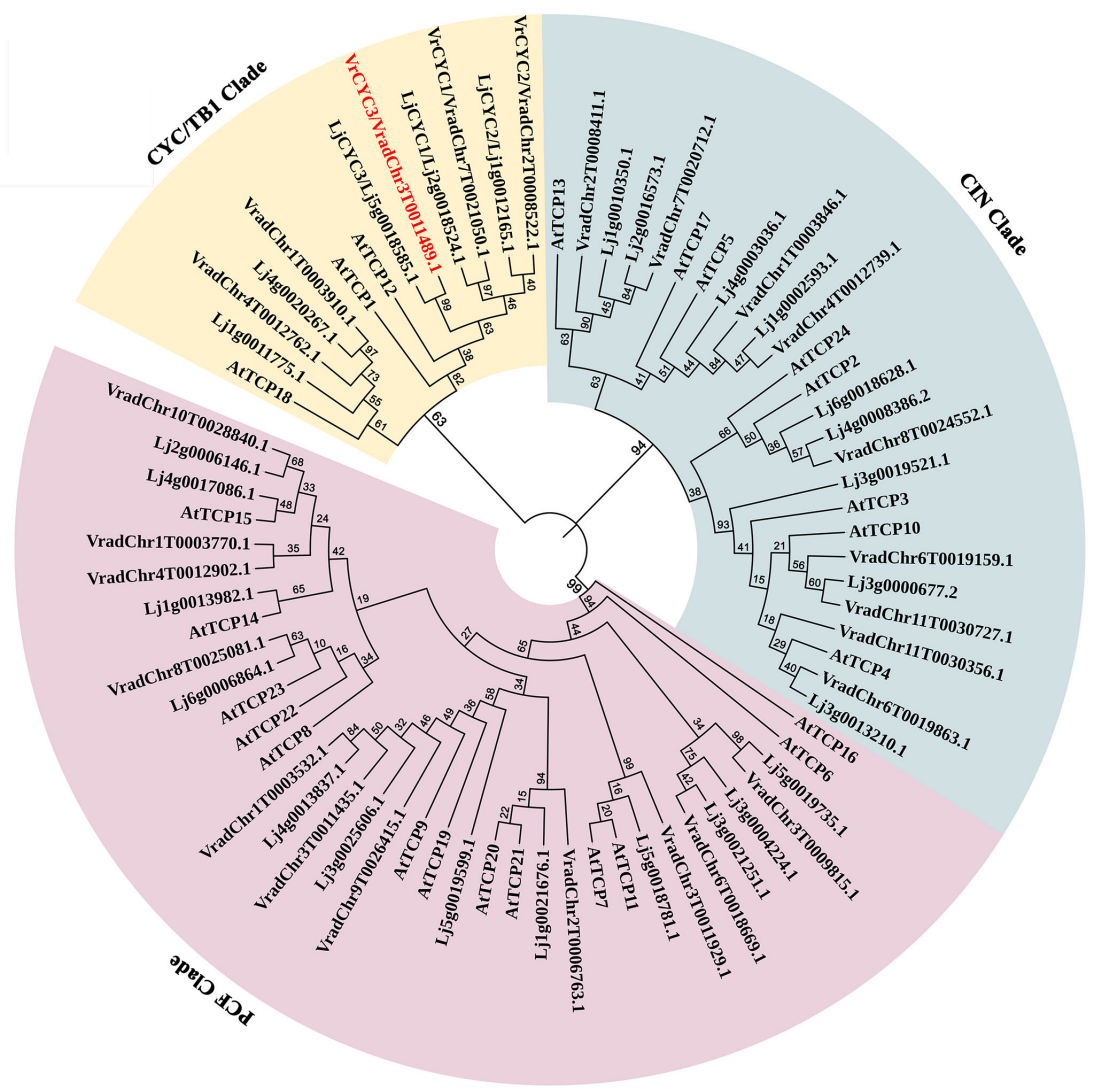
Phylogenetic analysis of TCP family proteins in mungbean. The phylogenetic tree was generated using the MEGA7.0 program and displayed by iTOL. The TCP family proteins from *Vigna radiata*, *Lotus japonicus* (Feng et al., 2006; Wang et al., 2008) and *Arabidopsis thaliana* were used. Different clades were indicated in a specific background colour. VrCYC3 was shown in red font.

Sequence analysis indicated that *VrCYC3* contained two exons and one intron and encoded a 375-AA peptide (**Figure 3**, **Figure 4**). We conducted a BLASTP search for sequences with homology to VrCYC3 in our mungbean database. It was found there were 25 TCP family proteins in mungbean M5311 genome (**Supplementary Table 2**), which were classified into three clades by phylogenetic analysis with 26 TCP family proteins in *L. japonicus* (*Lj1.0v1*; Li et al., 2020) and 24 TCP family proteins in *Arabidopsis* (**Figure 5**). Results showed that VrCYC3 had another two close related homologs VradChr7T0021050.1 (VrCYC1) and VradChr8T0024552.1 (VrCYC2), which also belong to the ECE CYC2 clade (Howarth and Donoghue, 2006; Zhao et al., 2019), together with VradChr1T0003910.1 and VradChr4T0012762.1, forming the CYC/TB1 clade of the TCP family proteins in mungbean (**Figure 5**).

### Expression and localization of VrCYC3

**Figure 6:**
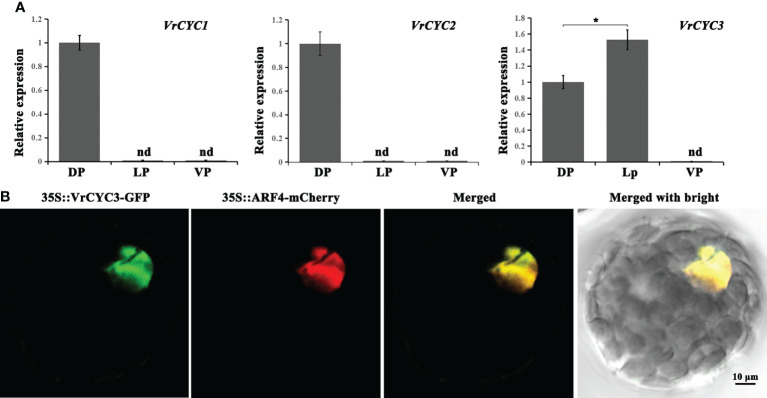
Gene expression of *VrCYCs* and subcellular localization of VrCYC3. **(A)** Relative expressions of *VrCYC1*, *VrCYC2* and *VrCYC3* in the dorsal petal (DP), lateral petal (VP) and ventral petal (VP) of WT. The data were means ± SD. nd, not detected. The Student’s test was used. *p < 0.05. **(B)** Subcellular localization of VrCYC3-GFP in mungbean protoplasts. OsARF4-mCherry was the nuclear marker. It was analyzed for green fluorescence emission, mCherry fluorescence emission and bright-field illumination.

The expression patterns of three *CYC*-like genes in three types of petals of the wild type plants were analyzed by qRT-PCR. We found that both *VrCYC1* and *VrCYC2* were only expressed in the dorsal petals, whereas the expression of *VrCYC3* could be detected in both dorsal and lateral petals, but with a relative higher level in the lateral ones (**Figure 6A**).

To investigate the subcellular localization of the VrCYC3 protein in mungbean, full length of the *VrCYC3* ORF was fused with the report gene GFP and then the fused construct was transiently expressed in the mungbean protoplasts through PEG4000-meidated transformation. Results showed that VrCYC3-GFP fluorescence was associated with the nucleus in the protoplast, merging with the signal of the positive control OsARF4-mCherry (Shen et al., 2010; **Figure 6B**).

### Protein interaction between VrCYC3 and its related homologs in mungbean

**Figure 7:**
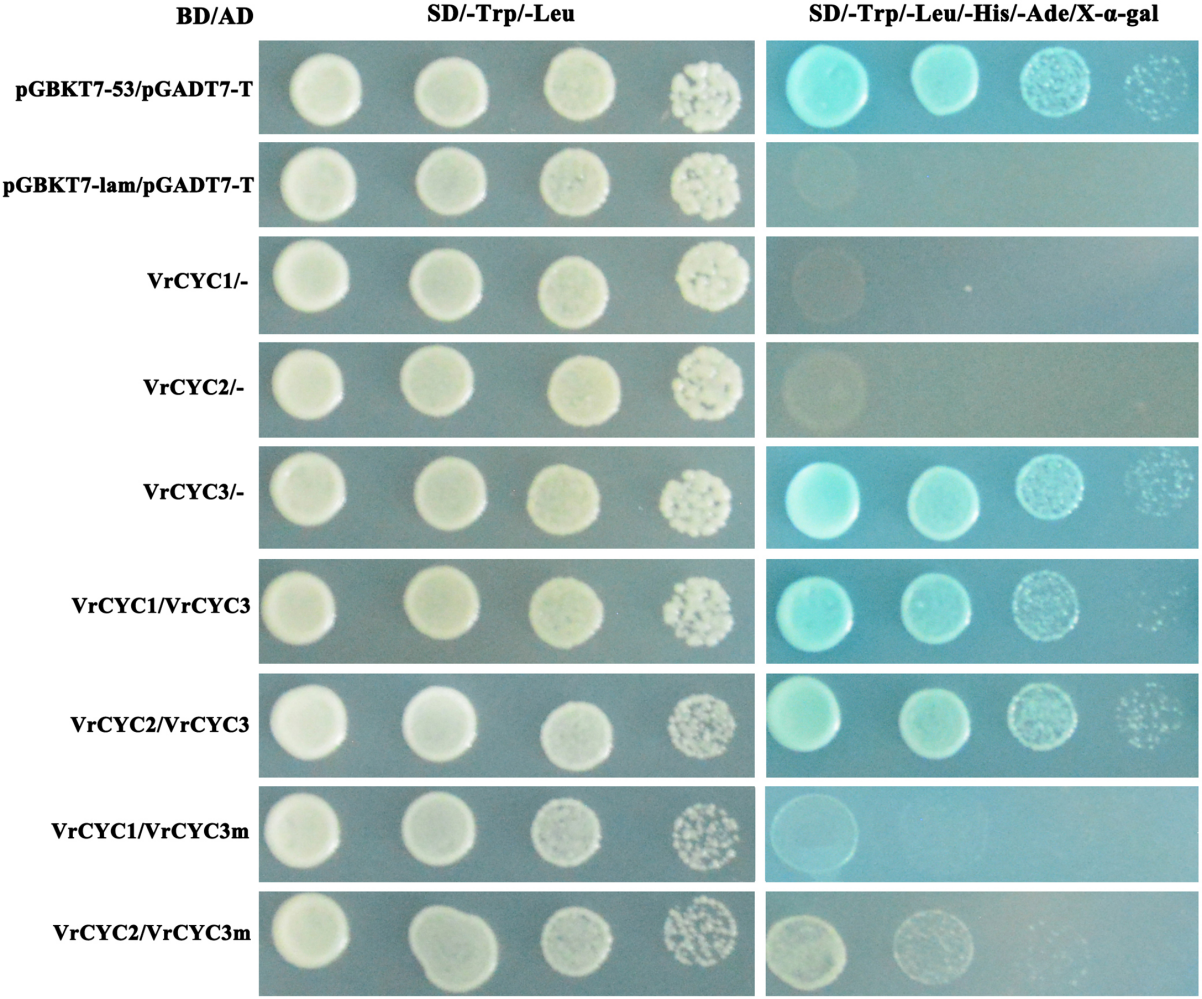
VrCYC3 interacted with VrCYC1 and VrCYC2 in yeast. AH109 cells were co-transformed with GAL4 BD fused VrCYC and empty GAL4 AD (-) or GAL4 AD fused VrCYC. VrCYC3m, a mutated VrCYC3 form lacking two residues, Argnine (R) and Alanine (A), in R domain as in *kw-2* mutant. Transformants were spotted on the plates with SD/-Trp/-Leu and SD/-Trp/-Leu/-His/-Ade/X-α-gal in 1-, 10-, 100-, and 1000-fold dilutions. The vectors of pGBKT7-53 and pGBKT7-lam, co-transformed with pGADT7-T, were used as the positive and negative control in the assays, respectively.

It has been previously reported that the CYC-like proteins form dimer to control petal development in *L. japonicus* (Xu et al., 2016; Yuan et al., 2020). To test the possibility of the interactions among three CYC-like proteins in mungbean, the GAL4 binding domain (BD) and activating domain (AD)were fused with different VrCYC proteins. The interactions between VrCYC3 and VrCYC1, VrCYC3 and VrCYC2 were analyzed in yeast two-hybrid system respectively (**Figure 7**). The assays showed that VrCYC3 interacted with VrCYC1 and VrCYC2 *in vitro*. Moreover, it was found that the loss of two amino acid residues in the R domain of VrCYC3 significantly affected its interaction with VrCYC1 and VrCYC2, suggesting that the R domain could play a role in protein-protein interaction.

In addition, the results also showed that the VrCYC3 protein exhibited high expression of the reporter gene (**Figure 7**), suggesting that VrCYC3 had strong transcription activating activity. Conversely, the VrCYC2 and VrCYC1 fusion proteins with GAL4 BD did not exhibit this activity in the assays (**Figure 7**).

## Discussion

In this study, two *kw* mutants (*kw-1* and *kw-2*) with centralized lateral petals were isolated by screening the large-scale gamma ray mutagenesis population about 70,000 M_2_ lines in mungbean, a species with asymmetric flower. Interestingly, the mutations also allowed each left ventralized lateral petal to form a ectopic spur, which developed normally on the left ventral petal in the wild type plant (**Figure 1**). It was found that *KW* gene encoded the TCP family protein VrCYC3 in mungbean, the ortholog of LjCYC3 and PsCYC3. All of these genes fall into the CYC2 clade of ECE TCP genes, and not the CYC3 clade, which has been lost in Papilionoideae (Howarth and Donoghue, 2006; Zhao et al., 2019). Our previous studies have shown that in the Papilionoideae with zygomorphic flower including *L. japonicus* and pea, loss of function of the orthologs of *LjCYC3/VrCYC3* lead to the ventralized petals at the lateral position (Feng et al., 2006; Wang et al., 2008). Thus, our results suggested that LjCYC3/VrCYC3 orthologs have functional conservation in determining the lateral petal shape and identity of flowers with zygomorphy as well as asymmetry in Papilionoideae. Consistently, it has been proposed that the asymmetric flower in some lineages in *Vigna* is the derived form of zygomorphy in Papilionoideae (Endress, 2001; Tucker, 2003). In addition, in mungbean flower, the three types of petals are internal (IN) asymmetric. However, the loss of function of VrCYC3 does not show the defective IN asymmetry, suggesting that there are other factors independently determining IN asymmetry in mungbean.

The establishment of floral zygormorphy is controlled by a module that relies on regulatory interaction among CYC-like TCP proteins and MYB proteins such as RADIALIS(RAD) and DIVARICATA (DIV) in the model plant *A. majus* (Raimundo et al., 2013). Recently, several studies suggested that the MYB transcription factors of this module have potential conserved function in the flower symmetry in other plants such as the Orchid and Lamiales (Valoroso et al., 2019; Sengupta and Hileman, 2022). In Asteraceae, the CYC-like TCP genes are required for the flower type specification (Broholm et al., 2008; Fambrini et al., 2011; Chapman et al., 2012; Juntheikki-Palovaara et al., 2014; Garcês et al., 2016; Huang et al., 2016; Ding et al., 2020; Sun et al., 2022). Interestingly, it is found that the MADS-box protein GRCD5 activates *CYC*-like gene expression during flower development in *Gerbera hybrida*, revealing an interplay between CYC-like TCP protein and MADS-box protein in the control of the flower symmetry in Asteraceae (Zhao et al., 2020; Yu, 2020).

However, in Papilionoideae such as pea and *L. japonicus*, none of MYB family transcription factors has been found to control flower symmetry. So far, the upstream and downstream targets or interacted partners of CYC-like TCP proteins involved in the dorsal-ventral patterning were not identified in Papilionoideae, except that a protein kinase LjRLK1 is found to interact with and modify CYC-like proteins in *L. japonicus* (Xu et al., 2017). In this study, we found that in the *kw-2* mutant, the ventralization of lateral petals was not complete, compared with *kw-1* mutant. Sequences analysis showed that a 5-bp deletion in *kw-1* would lead to a frameshift and premature stop codon, lacking most parts of VrCYC3 protein (**Figure 4**), while the 6-bp deletion in *kw-2* mutant would result in the translation of a mutated VrCYC3 protein lacking two residues in R domain which is predicted to form a coiled coil mediating protein-protein interaction (Cubas et al., 1999). Consistently, we found that the mutation in R domain decreased the affinity of the interaction of VrCYC3 with VrCYC1 and VrCYC2. Taken together, *kw-2* should not be a null mutant, which could be used for the secondary mutagenesis to screen enhancers or repressors and then to identify key components involved in the CYC pathway to regulate flower symmetry in Papilionoideae.

## Data availability statement

The original contributions presented in the study are included in the article/**Supplementary Material**. Further inquiries can be directed to the corresponding author.

## Author contributions

XL, MS, YJ, DQ, and QP performed experiment. XL and LZ analyzed the data and prepared the manuscript. All authors contributed to the article and approved the submitted version.

## Funding

This research was funded by the National Natural Science Foundation of China (grant no. 31200179) and the Jiangsu Agricultural Science and Technology Innovation Fund of China (CX (20)3030).

## Conflict of interest

The authors declare that the research was conducted in the absence of any commercial or financial relationships that could be construed as a potential conflict of interest.

## Publisher’s note

All claims expressed in this article are solely those of the authors and do not necessarily represent those of their affiliated organizations, or those of the publisher, the editors and the reviewers. Any product that may be evaluated in this article, or claim that may be made by its manufacturer, is not guaranteed or endorsed by the publisher.

## Supplementary material

Supplementary Figure 1 Alignment of CYC1/2/3 proteins in *Vigna radiata*, *Lotus japonicus* and *Pisum sativum*. Red line, TCP domain; Blue line, R domain.

Supplementary Table 1 Primers used in this study.

Supplementary Table 2 Annotated genes in the *kw* mapping region of mungbean M5311 genome.

Supplementary Table 3 TCP family proteins from *Vigna radiata*, *Lotus japonicus* and *Arabidopsis thaliana*.
