# Supplementary figures and images for: Genetic control of the lateral petal shape and identity of asymmetric flowers in mungbean (Vigna radiata L.)
Source: Front Plant Sci. 2022 Sep 29;13:996239. doi: 10.3389/fpls.2022.996239 (PMC9560771; doi:10.3389/fpls.2022.996239)

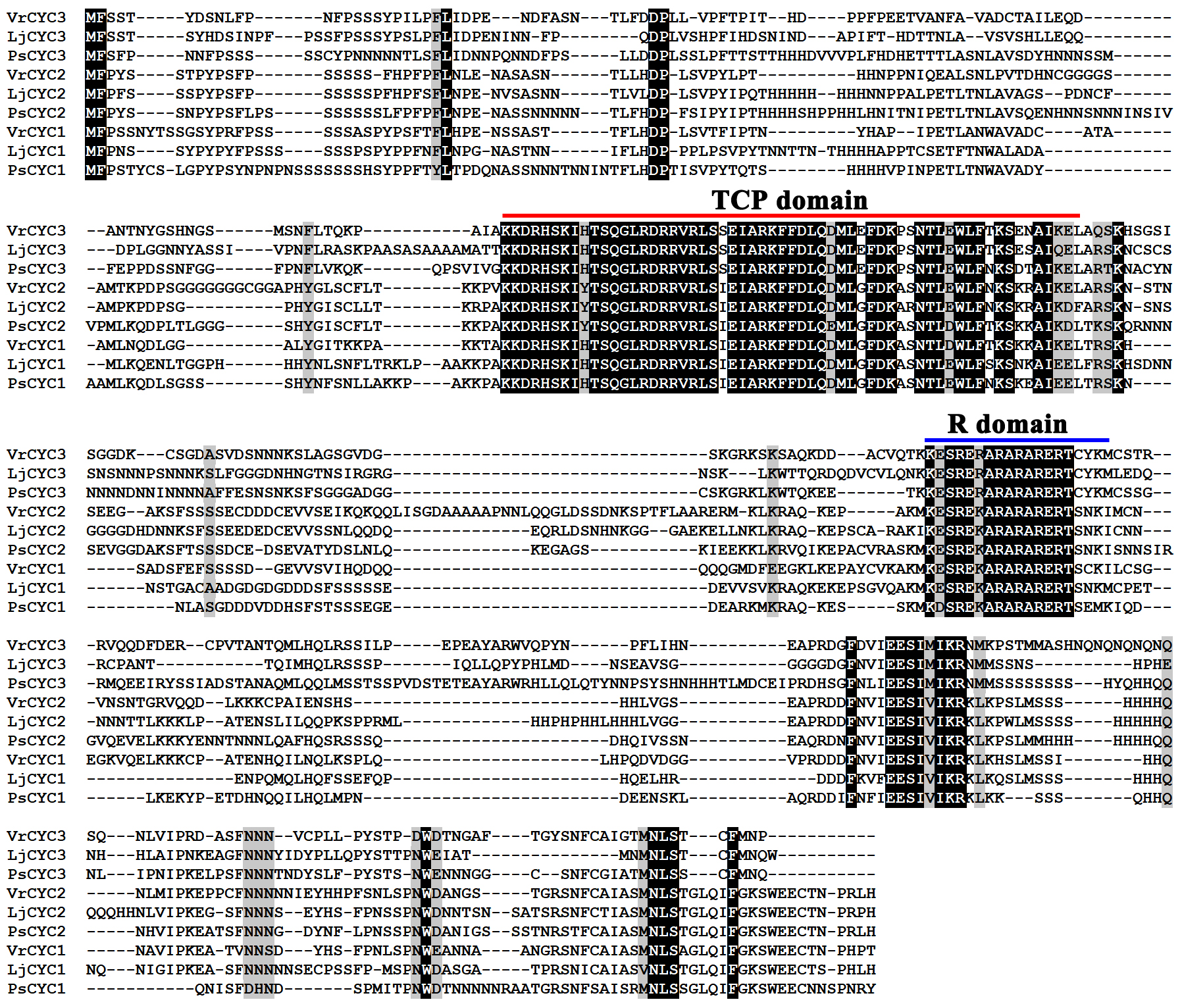

Supplement: Supplementary Figure 1 — Alignment of CYC1/2/3 proteins in Vigna radiata, Lotus japonicus and Pisum sativum. Red line, TCP domain; Blue line, R domain. [file Image_1.jpeg]
